# DEALING WITH A DISTRESSED SPOUSE: MARITAL DYNAMICS IN SAME-SEX AND DIFFERENT-SEX COUPLES

**DOI:** 10.1093/geroni/igz038.1531

**Published:** 2019-11-08

**Authors:** Debra Umberson

**Affiliations:** University of Texas, Austin, Texas, United States

## Abstract

We use a mixed-methods strategy to generate insights into gendered marital dynamics when one partner is experiencing high levels of psychological distress/depression. Our data are unique in their dyadic design and in the inclusion of same-sex and well as different-sex marital dyads. The data are from closed- and open-ended survey responses (from 808 gay, lesbian, and heterosexual spouses in 404 unions) as well as in-depth interview data (with a subsample of 45 gay, lesbian, and heterosexual couples in 90 unions). Respondents were asked about their most significant period of emotional distress during the marriage, how their spouse reacted to their distress (e.g., providing emotional or instrumental support, withdrawing), and how much they worried about burdening their spouse. Respondents were also asked how they reacted to their spouse’s periods of emotional distress. Preliminary results point to gendered experiences of distress within marriage that sometimes differ for same-sex compared to different-sex couples.

